# Challenges arising for older workers from participating in a workplace intervention addressing work ability: a qualitative study from Germany

**DOI:** 10.1007/s00420-020-01639-x

**Published:** 2021-01-24

**Authors:** Khira Sippli, Pia Schmalzried, Monika A. Rieger, Susanne Voelter-Mahlknecht

**Affiliations:** 1 Institute of Occupational and Social Medicine and Health Services Research, University Hospital Tuebingen, Wilhelmstraße 27, 72074 Tübingen, Germany; 2 Institute of Occupational Medicine, Charité-Universitätsmedizin Berlin, Corporate Member of Freie Universität Berlin, Humboldt-Universität zu Berlin, and Berlin Institute of Health, Augustenburger Platz 1, 13353 Berlin, Germany; 3 Institute for Applied Economic Research at the University Tuebingen, Schaffhausenstr. 73, 72072 Tuebingen, Germany

**Keywords:** Aging workforce, Aging, Older worker, Work ability, Workplace intervention

## Abstract

**Objective:**

Studies examining what renders workplace interventions to sustain and promote work ability of older workers successful have largely neglected older workers´ perspective. This paper outlines the results of a study with regard to older workers´ experiences and expectations of a workplace intervention. Based on these findings, some reflections on how to improve the design and the implementation of workplace interventions for older workers are provided.

**Methods:**

Semi-structured interviews were conducted with older workers (*N* = 8) participating in a workplace intervention undertaken at one production site of a large manufacturing company in Baden-Wurttemberg/Germany. The interview guide included questions on participants´ experiences with and expectations of the intervention. The interviews were recorded, transcribed verbatim and analyzed using qualitative content analysis according to Mayring (2014).

**Results:**

Older workers´ reported some challenges they face due to their participation in the workplace intervention. These resulted from the work environment (physical challenges), the work process design (new long work cycle), the work organization (tight time allowances, little job rotation, change of teams, age stereotypes) and the management of the workplace intervention (bad information, feeling of occupational insecurity and lack of being valued).

**Conclusions:**

The study shows that challenges arising for older workers from their participation in the workplace intervention may have counteracted the promotion of work ability. As findings suggest, some of these challenges might have been avoided either by considering workers´ perspective during design and implementation of an intervention or by referring to evidence on aging and work ability.

**Supplementary Information:**

The online version contains supplementary material available at 10.1007/s00420-020-01639-x.

## Background

Demographic change, i.e., a decrease in birth rates and a parallel increase of life expectancy, induces a continuing aging of many societies. To sustain the pension system, welfare states have carried out reforms which prolong working lives by delaying retirement age. For companies, demographic change and policy reform imply that they increasingly need to recruit and retain older workers to meet their personnel requirements.

Concerns remain whether older workers can work longer. The work force participation of older workers is still low, implying that workers often leave work before reaching retirement age. Thus, companies need to take actions to enable workers to remain in the workforce until retirement age.

One popular approach to increasing retention rates of older workers is to adapt work demands to their needs. The concept of work ability as developed by the Finnish Institute of Occupational Health (Tuomi et al. 1991; Ilmarinen 1997) represents this idea. Work ability is defined as a balance between work demands (work environment, contents, demands, organization of work and management) and individual capacities (health and functional abilities, competence and values) (Ilmarinen 2009).

Work ability, i.e., the balance between work demands and individual capacities, needs to be promoted, because a poor balance between work demands and individual capacities means that a worker might not be able to meet work demands in physical, mental and mixed work (Ilmarinen et al. 1997; Ilmarinen 2009). If persistent over time, a poor balance between work demands and individual capacities might even cause individual capacities to decrease, e.g., by causing health impairments, and thus making work ability decrease further.

Starting points for establishing the balance between work demands and individual capacities are summed up by the “work ability- house model” (Gould et al. 2008). The work ability house model is an advancement of the concept of work ability and displays a number of factors which are causally related to work ability and can be addressed for work ability promotion. The main factors that can be addressed to promote work ability are health, competence, values and work. Besides, the context of work, e.g., family, social networks or institutional regulations, is important for work ability as the house model illustrates.

According to Ilmarinen (2009), work ability should be constantly promoted during work life to prevent early retirement. However, promotion of work ability seems to be particularly important during aging. When measured by the work ability index (WAI), on an average of all workers, work ability declines with age, while the between-individual variance of work ability increases (Ilmarinen 2009). As an extensive literature suggests, this result is due to a decline in individual cognitive and physical capacities that comes with aging and an occasional or permanent exposure to adverse working conditions.

With regard to older workers´ cognitive capacities, literature suggests that aging has a negative impact on various cognitive functions, e.g., on the ability to retain multiple items in the working memory, and the speed of processing information (Borella, Carretti, & De Beni 2008; Johnson et al. 2011). By impacting postural stability, an attention decrease that comes with ageing increases the risk for loss of balance and falls when moving and performing cognitive tasks simultaneously (Brown et al. 1999; Bernard-Demanze 2009).

With regard to older workers´ physical capacities, the body of literature suggests that these start to decrease at the age of 40 and that this decrease becomes marked after the age of 50 (Donato et al. 2003; Kenny et al. 2008). As a consequence, the physical work capacity of a 65-year-old worker could be reduced by up to 50% as compared with an average 25-year-old worker (Ilmarinen and Rantanen 1999). Some main physical capacities that decline are muscular strength and endurance (Ostchega et al. 2004); balance, flexibility, coordination, and agility (Fujiyama et al. 2012; Kenny et al. 2008; Marques et al. 2016; Ostchega et al. 2004); as well as sensory abilities, i.e., eyesight, hearing (Silverstein 2008).

The decrease in physical capacity usually exceeds the decline in mental or social abilities (Chan et al. 2000; Ilmarinen 2001). In addition, workers in physically demanding jobs are more likely to experience cognitive decline as the review by (Fisher et al. 2017) shows. Thus, work ability of workers in physically demanding occupations might be more affected by ageing (Ilmarinen 2002).

Furthermore, as a result of the decline of different cognitive and physical capacities, the general risk of injury of older workers is increased (Bernard-Demanze et al. 2009; Blatteis 2012; Brown et al. 1999; Chan et al. 2000; Kenny et al. 2016a; Lamarche et al. 2017; Larose et al. 2013; McGinn et al. 2017; Meade et al. 2015, 2016; Silverstein 2008; Stapleton et al. 2014, 2015; Zwerling et al. 1998), particularly when there is a mismatch between workers´ physical abilities and job demands (Chan et al. 2000; De Zwart et al. 1996; Fraade-Blanar et al. 2017; Garg 1991; Ilmarinen et al. 1994; McCaughey et al. 2013; Pransky et al. 2005; Silverstein 2008; Wegman 1999).

In addition, older workers are at higher risk of suffering from chronic diseases which might have a major impact on work ability, productivity, and absence (Leijten et al. 2014) as well.

However, in the literature, it is known that the amount of the decrease of cognitive and physical capacities due to ageing is always dependent on the individual state of health and physical fitness (Leyk et al. 2010; Loeppke et al. 2013). Also, there is no consistent negative relationship between ageing and work performance (Kenny et al. 2016a; Silverstein 2008; Truxillo et al. 2015).

With regard to the impact of occasional or permanent exposure to adverse work demands on the decline of work ability in old age, the literature identifies some factors that might pose a risk. These include, for example, shift and night work (Costa 2005; Costa and Di Milia 2008; Costa and Sartori 2007), as well as chronically high physical demands throughout work life which are correlated with a strong decline in physical capacities (Savinainen et al. 2004) and a higher likelihood to develop musculoskeletal disorders (MSD) or to report musculoskeletal injuries (Nygard et al. 1988; De Zwart et al. 1996). A recent study found that physical, emotional, mental, and psychological job demands, as well as well the extent of social support at work predict the development of multi-site musculoskeletal pain (Oakman et al. 2017).

### Workplace interventions to promote work ability

As the “work ability house model” (Gould et al. 2008) illustrates, the work ability of older workers can be promoted by addressing either individual capacities (health, competences or attitudes) or work demands (work environment, work organization, management). As can be seen from the literature, workplace interventions with different designs can be undertaken for this purpose.

Work ability interventions that addressed older workers´ individual capacities to deal with work demands aimed at improving health, lifestyle and well-being at work. These interventions comprised a variety of measures for different groups of population, for example physical exercise (Mackey et al. 2007; Rutanen et al 2014), ergonomic training (Karazman et al. 2000), selection, optimization and compensation (SOC) training (Muller et al. 2016) and counselling and education (de Boer et al. 2007).

Work ability interventions for older workers that addressed work demands encompassed the adaptation of the work environment, work organization and/or working conditions to reduce work-related stress or physical demands. These interventions undertaken involved ergonomic adaptations, i.e., a redesign of job tasks, tools, and environment (Andersson-Felé 2005), ergonomic workstations (May et al. 2004), reduction of unfavorably designed shift work (Härmä et al. 2006), flexible work arrangements (Morelock et al. 2017), reduction of working hours and exemption of older workers from nightwork (van der Meer et al. 2016) as well as improvement of the working climate by means of problem-solving techniques (Koolhaas et al. 2015).

### Effectiveness of work ability interventions

Both from an employer´s and a worker´s perspective, it is relevant that workplace interventions undertaken are effective with regard to sustaining and promoting work ability.

However, meta analyses and reviews of the existing literature show that it is unclear whether workplace interventions are effective with regard to improving work ability of workers in general and older workers in particular (Cloostermans et al. 2015; Crawford et al. 2010; McDermott et al. 2010; Poscia et al. 2016; Oakman et al. 2018; Tarro et al. 2020). This is due to low methodological quality, heterogeneity, and paucity of studies on work ability interventions, particularly for older workers, implying that findings are weak, not generalizable and inconsistent (Cloostermans et al. 2015; Crawford et al. 2010; McDermott et al. 2010; Poscia et al. 2016; Oakman et al. 2018; Tarro et al. 2020).

Furthermore, there is a lack of knowledge about which characters of workplace interventions for older workers, e.g., their design, context, or implementation, render them effective with regard to desired outcomes (Tarro et al. 2020). This appears to be a consequence of paucity, low methodological quality and heterogeneity of existing studies as well. However, as Oakman et al. (2018) points out, the complexity of work ability also makes it challenging to measure the effectiveness of workplace interventions to promote work ability of older workers.

Complexity of work ability is also a challenge for the implementation of workplace interventions (Oakman et al. 2018; Ilmarinen 2019). As Ilmarinen (2019) points out, knowledge about what factors should be addressed to promote work ability is growing while it is not clear how to translate it into action. Thus, more attention should be paid to implementation of workplace interventions to promote work ability.

Up to now, workers´ perspective on workplace interventions to promote work ability of older workers has been largely neglected by the literature. This article aims to contribute to the literature by delineating how older workers perceived the design and implementation of a workplace intervention they participated in. Thereby, insights can be gained into what challenges may arise for older workers from their participation in workplace interventions modifying work environment, work conditions, and/or the work process to promote work ability. These insights may assist practitioners in improving the future design and implementation of workplace interventions by reducing possible negative consequences for older workers arising from their participation. Thus, such interventions probably could be rendered more effective with regard to promoting older workers´ work ability.

## Methods

### Study design and sample

The study was first conducted as a master’s degree project in Sociology by one author (PS), who was supervised by the study initiator (SV-M). The present manuscript summarizes the results of a re-evaluation of the interviews with a different research focus.

The objective of the study was to explore older workers´ experiences with a workplace intervention implemented by a manufacturing company. As a standardized survey would not have been able to analyze individual experiences in depth and the overall number of participants in the intervention was low, a qualitative study design was chosen, i.e., it was planned to conduct semi structured interviews with older workers participating in the intervention. These workers were to be recruited by the program supervisor, yet on a voluntary basis. Eight (*N* = 8) out of those older workers participating in the intervention at the time when the study was conducted gave their informed consent to be interviewed. All of them were included in the study.

With regard to sample characteristics (see Table 1 in the supplementary file), all interviewees were male, which reflects the overall gender relation of the work force of the manufacturing company. At the time of the interview, participants were on average 54 years old and had worked in the company for an average of 26 years.

To gather background information about the development of the intervention and to comprise more views on its implementation, it was planned to conduct further interviews with a company doctor, someone responsible for the intervention from the human resources department of the company, and a member of the work council of the company. However, as the company withdrew its consent for conducting interviews with the company doctor, the person from the human resources department, and the member of the company’s work council for unknown reasons, these interviews could not be realized.

### Data collection and setting

Based on the research interest and according to standard procedure three interview guides were developed for participating workers, company doctor and program supervisor. The interview guide for workers participating in the intervention contained open questions to prompt narration as well as follow-up questions to enquire about workers´ expectations and experiences of the implementation of the workplace intervention (see Table 2 in the supplementary file).

Qualitative semi-structured interviews with participants of the intervention (*N* = 8) were conducted by one interviewer (PS) in October 2014. The interviews occurred at the workplace during working hours and lasted approximately 1 hour each. With the consent of the interviewees, the interviews were audio recorded.

As already mentioned, interviews with a company doctor and the program supervisor could not be conducted as the company withdrew consent.

### Data analysis

The conducted interviews were pseudonymized and transcribed verbatim by the interviewer (PS) herself in 2015. In the end of 2016, a qualitative content analysis of the interviews according to Mayring (Mayring 2014) was conducted. Content analysis is a transparent and comprehensible method for the analysis of qualitative data as it consists of a number of fixed steps (category creation, coding and data analysis) with the objective of reaching higher levels of abstraction from the data.

In the present study, categories (“codes”) were derived inductively from the material of two interviews by summarizing the texts and identifying key issues that were then labelled as main categories or sub-categories as agreed upon by two researchers (KS, SV-M). These categories were tested on text passages of one interview. Then, main and sub-categories were attributed to text passages of all eight interviews. This “coding” was conducted by one researcher (KS) using MAXQDA 11 plus (Verbi Software Comp., Berlin, Germany), which is a software that helps researchers to structure and systematically analyze large amounts of qualitative data. The coding was then discussed by two researchers (KS, SV-M) who have a professional background in sociology (KS) and occupational medicine (SV-M), respectively, to achieve intersubjective validity of results. Finally, passages of text attributed to each code were analyzed.

### Description of the workplace intervention investigated

In the beginning of 2014, the management of a production site of a global manufacturing company in Baden-Wurttemberg/Germany undertook an effort to meet the challenge of its aging workforce. Aiming to lower work demands for older workers with and without impairments and thus to improve work ability, a workplace intervention was undertaken. This workplace intervention addressed a maximum number of 220 highly qualified blue-collar workers aged 53 and above, as well as blue-collar workers with work-related impairment(s) or on long-term sick leave. In addition to age, impairment, or long-term sick leave, a further requirement for participation was the affiliation to a certain wage group related to the workers´ qualification. Participation was voluntary and some workers were granted the opportunity to return to normal production if they were not satisfied with the intervention.

Initially, from all applications, 80 workers were selected for participation in the workplace intervention. Of these, 24 workers were aged 53 and above, while 24 workers were in the active phase of part-time work for older employees. In addition, 32 workers with impairments participated, of whom 12 workers were no longer able to work on an assembly line under a given work cycle. Further workers joined the intervention shortly after. The qualitative evaluation was performed approximately 6 months after the start of the intervention.

The main component of the workplace intervention was to adapt the work process by prolonging the work cycle as compared to normal production. Additionally, the assembly line was turned off during one work cycle, so that workers could complete their tasks without leaving their work station.

Furthermore, several ergonomic adaptations of the work environment were envisaged. For workers with work-related impairments, extra workstations should be built, which, for example, demanded less strength during work steps. However, it is unclear to what extent ergonomic adaptations had been realized at the point in time when interviews were conducted.

The workplace intervention was implemented outside of normal production in a separate production hall of the production site, and only participants of the workplace intervention worked there. Inside the production hall, a new production line was set up where a new product was manufactured in two-shift production in limited edition.

## Results

Interviews with participants in the workplace intervention outlined above revealed a number of challenges for older workers that arose from the implementation of the measure. Knowledge about these challenges can help improve prospective workplace interventions, and help employers choose the right organizational policy and practice for implementation.

Hereafter, an overview of identified challenges is given (see Table 3 in the supplementary file for an overview), and illustrating quotations are given in the specific sections below. As some workers were not German native speakers, the English translations of the quotation mimic the individual accents.

Results on challenges arising for older workers from their participation in the workplace intervention are presented in relation to the respective action taken by the company, whereas these are assigned to the dimension of the “work ability- house model” (Gould et al 2008) they address.

As outlined above, the workplace intervention addressed the dimension of work by implementing several measures for adapting work demand by adapting work environment, work equipment and work organization. These adaptations had unintended effects on other dimensions of the “work ability- house model” as well, particularly on the dimension of values which comprises work motivation.

### Work environment and work equipment: physical challenges arising from low automation and outdated work environment

For older workers participating in the workplace intervention, one challenge that arose was that the general work environment and the work equipment of the intervention were not adequate for their needs, thus imposing physical challenges.

First, workers reported that the equipment in the production hall was outdated. In comparison with other production halls, the degree of automation was reported to be considerably lower. At several workstations workers had to use their own physical strength for tasks that in normal production were taken over by robots or were facilitated by machines. For example, workers had to tighten screws manually at some workstations, whereas this working step was done automatically in high volume production. In some cases, machines for facilitating tasks existed, yet workers had to lift them up to be able to use them.

Workers also reported that there were no hydraulic lifts at the workstations. Thus, in contrast to normal production, workers had to bend or kneel down, step up or work overhead when exercising certain work steps as they could not fit the height of the assembly line to their own body size. One worker put it like this:

*“And here it’s um, you have to adjust [the body] to the material. There [in normal production] it was the other way around. You could adjust the material to your body. And that’s absolutely not possible here. Zip.” (MA8, 224)*

Motions like bending, kneeling or working overhead which were implied by work environment and work equipment were perceived as demanding for older workers, especially as sitting while working was not envisaged. Also, workers pointed out that the work equipment did not allow them to escape certain movement which they perceived as particularly difficult due to individual health problems such as back pain or knee problems, e.g., lifting or kneeling.

Thus, workers assessed both equipment and ergonomics of the workplaces as inadequate for older workers and particularly for workers with health constraints. Special ergonomic adaptations undertaken within the intervention were not mentioned by the interviewees, i.e., possibly not detected.

Second, workers had to move around more than in normal production as the assembly line was reported to be not running during the long work cycle. Thus, according to the assessment of workers interviewed, work was more physically challenging for them than in normal production.

*“So concerning, concerning physical work or the job itself it’s maybe a little worse than in large-scale production- because there were small- small – small movements and so. Here the amount is a little bigger and also the walking distances are a bit too far. It’s big. It’s a lot, you have to get that- get that material and and get the parts.” (MA6, 109)*

*“You have to – need more strength and have to move more, that um you notice that.” (MA1, 179)*

Third, workers criticized that the production hall where the workplace intervention was located was smaller than usual. This had implications for workflow at the different work stations of the assembly line. Workers reported that they felt constricted when working at work stations and that they often got in the way of their colleagues when, e.g., fetching tools for the next work step.

*“I mean things are pretty squished together here. While you work you hinder yourself, um I mean the colleagues hamper each other a lot, um, because they – they stand in each other’s way or two want to work at the same spot. And everything is super tight.” (MA1, 315).*

Finally, workers assessed the climate in the production hall as difficult to bear for older workers. Due to a lack of air-conditioning, the hall heated up quickly in summertime and cooled down in wintertime. Workers also reported that some work stations were badly illuminated what made certain tasks difficult to accomplish.

### Work process design: cognitive challenges arising from the introduction of a long work cycle

Another challenge for older workers participating in the intervention arose from the prolongation of the work cycle, i.e., the time allowance set for conducting a sequence of work steps at a work station. While the average duration of a production rhythm was one to one and a half minutes in normal production, the work cycle at work stations of the workplace intervention was between 16 and 18 min. Thus, participants had to conduct a considerably longer sequence of work steps at a work station during a work cycle than before. As the majority of older workers participating in the workplace intervention was used to working in a short work cycle, they perceived the adaptation to the prolonged work cycle, i.e., learning to remember a long sequence of work steps, as cognitively demanding and time consuming. Particularly, workers reported that cognitive demands had been very low in normal (large-scale) production where they had worked for years, because they had to repeat the same work steps monotonously.

*“In small-scale production, you don’t just do two wires, but you have to build the whole module. That means you can never just zone out, you have to concentrate more, because you have to, you have to stay with it. In large-scale production, you could just, yah you didn’t have to concentrate so much, because it was always just the same.” (MA6, 66)*

Thus, workers reported that they struggled with remembering work steps of a sequence and felt under high pressure not to make a mistake, as this would be more difficult to correct in a long sequence of work steps. One common strategy workers used was always to execute all work steps in the same sequence. Yet, interviewees reported that a number of coworkers were not able to memorize long sequence of work steps implicated by the long work cycle and thus returned to normal production.

### Work organization I: increased job demands induced by tight time allowances

A major challenge for participants was that the work process was designed in a way that created time pressure so that workers had difficulties in performing their work and perceived work demands as high. Thus, the majority of interviewees did not perceive the intervention as lowering job demands and/or facilitating work for older workers.

Workers reported that they felt largely under time pressure when conducting their job tasks due to preset time allowances. Several interviewees reported that they had difficulties to finish their work sequences in the given time allowance as they simply could not execute their tasks faster. Yet, it remains unclear whether workers´ problems with time allowances were due to individual work performance or due to miscalculated time allowances. Workers indicated the time allowances for work sequences at some work stations to fit better to time needed for their execution than others. This was ascribed to the fact that time allowances are commonly computed automatically in the company, and only afterwards adapted to practice; a process which workers assessed as non-transparent and not reasonable. The adaptation of time allowances to practice had not yet happened when workers were interviewed approximately 6 months after the start of the intervention. The interviewees reported that further reductions of time allowances for the same work sequences were planned by the management to increase production output per day.

To come to terms with time allowances, workers tried to work faster or to adapt work steps to save time. Some workers also reported that they tried to help each other with finishing tasks in time. However, this was the exception, as most workers struggled with time allowances themselves. Workers also tried to get help by calling the “supporter” (“Unterstützer”), but it often took very long for him to arrive.

The feeling of time pressure was enhanced by the fact that workers had little time to relax between single work sequences and that most tasks had been pre-commissioned, i.e., they just had to grip material, and not pick it up at some distance, the latter being assessed as offering some rest.

### Work organization II: low possibility for recovery due to a malfunction of job rotation

A further challenge for workers participating in the intervention was that job rotation did not function well, thus preventing the offer of relief.

Job rotation did not function properly due to personnel shortages caused by sick-leave and workers’ holidays. These shortages occurred from the beginning of the workplace intervention and hindered training the workers at work stations. Therefore, workers were only able to work at a limited number of work stations and flexibility for job rotation was low. Progress in training and increasing flexibility for job rotation was further hindered because workers with impairments who had a medical certificate documenting their inability to perform certain tasks were exempted from normal job rotation and only worked at selected work stations or in pre-commissioning. Under the prevailing conditions, interviewees assessed this practice to some extent as unfair treatment. For one reason, because it implied that workers without a medical certificate had to stay at the same work stations for unusually long periods. In addition, because it meant that workers without impairments had little chance to work at stations that were commonly assessed as easy and offering some recovery. Thus, some interviewees expressed their fear to be suffering from health problems in the long term if job rotation would persist to be not properly functioning.

*“And the other [not impaired workers], they just get [to do] what’s, what’s heavy or what’s not ergonomical. Yah, and eventually they also get impaired, that’s just the problem.” (MA6, 150)*

Several workers reported that they already suffered from pain after working at the same work station for longer periods of time, and that their pre-existing individual health problems were exacerbated by the malfunction of rotation. Hence, they had sympathy for special treatment of workers with impairments, but only to the point where their own well-being was endangered by overstrain. Interviewees reported that the malfunction of job rotation caused dissatisfaction and induced tensions in teams, causing the general working climate to suffer.

Several workers reported that they had tried to implement informal rotation in their teams to help themselves. However, the functioning of informal rotation was hampered by the same problem as job rotation, namely that most workers had only learned a rather limited number of work stations.

However, it remains doubtful if a better working job rotation could have offered sufficient relief for workers suffering from overstrain and pain, as in general there was little alternation between work stations with regard to motions to be made during work. The long work cycle implied that complex motion sequences involving lifting, kneeling, bending or else had to be made at all work stations.

### Work organization III: change of teams and social status

Another major challenge that arose for the majority of older workers participating in the intervention was a need to relocate and change their social work environment. Except for one interviewee, whose entire former team participated in the intervention, interviewees reported that they relocated alone, i.e., without their colleagues from their former teams. Most interviewees had not changed their workplace and their team for years, although internal changes were reported to be common inside the company. Thus, the vast majority described their participation in the intervention as socially demanding, as they had to integrate into a new team, i.e., learn how to communicate and deal with each other for cooperation to work.

However, integration into the new team was harder for some workers than for others. Some workers reported that it did not help them much that they had decided to participate in the intervention and could prepare mentally for the change, or the fact that everyone was in the same situation: they felt that they had to start completely new.

*“Um, many colleagues say: I think I’m starting first grade again […] like when you have your first day in a new company again, that’s how it is in principle. And you have to process that first.” (MA8, 26)*

Several interviewees also related their perception of the difficulty of changing their social environment to their age. Adapting to a fully new (social) work environment was perceived to be much harder for older workers than for young ones. For illustrating this experience, one worker referred to the common proverb *“You cannot shift an old tree without it dying.”* Thus, some interviewees expressed that they hoped this change of team would be the last before their retirement so that they would not have to start again from zero.

*“I'm still a nothing again, zero- don’t know anyone again, and of course that's another problem.” (MA8, 169)*

Another related challenge for many interviewees was the change of the social status they had in their former teams, which was connected to their position. All interviewees reported that they had held the position of group spokesman (“Gruppensprecher”), supporter (“Unterstützer”), or even deputy master craftsman (“Meisterstellvertreter”) in their former team. As such, they had had special responsibilities, but also a special social status. From the interviews it became clear that all interviewees were proud of this achievement and thus wanted to keep their position, as the following citation illustrates:

*“Yah okay, it was important for me uh to keep working as deputy master craftsman, ya know, […] you’ve somehow earned it and it was pretty uh important for me to have that again.” (MA7, 160)*

However, only half of the interviewees retained their position while participating in the intervention. Those who lost their position reported that they had found their former responsibilities to be stressful, but also interesting, and mourned the loss of their position as a degradation:

*“The work is, from one day to the next, that what you've done for years, what you've qualified yourself for, you've done training, you've done everything, and from one day to the next it doesn't count anymore.” (MA8, 26)*

However, it remained unclear from the interviews if this change of position and associated social status affected workers´ overall work motivation.

### Work organization IV: need for counter-acting age stereotypes invoked by the design of the intervention

Another challenge for participants was that the overall conception of the intervention invoked age stereotypes they had to deal with.

Interviews showed that the design of the work environment and the work process made participants aware of an age-related decline in physical and cognitive capacities and thus evoked the need for them to counteract age stereotypes. This was because older workers had problems fulfilling job demands within the set time allowances, yet noticed that younger workers had fewer or no problems with the requirements. These younger workers were increasingly participating in the intervention to compensate personnel shortages that partly occurred because older workers´ dropped out of the intervention due to overstrain or sick leave.

Another related consequence was that several workers felt a need to distance themselves from their age group to counter stereotypes and thus warrant their own work ability. They emphasized that they were physically and mentally more capable than their co-workers of the same age who had problems fulfilling job demands due to individual impairments. Interviewees only indirectly mentioned their own work impairments.

*“Of course that’s physical [strenuous], of course. Because at a certain age you also start to decline, I’m still relatively healthy – I’m 56 years old now. Still pretty fit, there are other people who aren’t so healthy.” (MA8, 113)*

In addition, they also mentioned that management spread age stereotypes regarding the higher capability of younger workers:

*“So you hear the example again and again, yes, a 25-year-old just has a bit more speed or uh more than a 55-year-old. You hear that again and again as an example. You can, when it comes to doubling the workload, it comes to how you feel in the work station, when you say you're at the limit here, so it's always mentioned as an example; ha, a 25 year old does that, and with a 55 year old you can't demand it like that anymore.” (MA1, 392)*

However, in their effort to counteract age stereotypes, interviewees also reinforced them. When asked what strengths and weaknesses older workers have. Strengths they attributed to older workers were experience and knowledge, self-confidence, high motivation, as well as relaxed dealing with stressful situations. Weaknesses they attributed to older workers were decreasing cognitive and physical abilities, such as MSD, lowered agility, and slowness in movements. However, interviewees also expressed their awareness of differences in physical and cognitive abilities between workers of the same chronological age. In this context, some workers also mentioned that with increasing age they found it more difficult for themselves to execute certain activities and even suffered from pain while working.

### Management I: bad information about the intervention causing disappointment

Another challenge for workers participating in the intervention was that they felt they learned about the features of the intervention only while participating. Hence, they complained about a bad information policy by the company and expressed that the intervention fell short of their expectations, also with regard to general age management by the company.

In short, older workers had expected that work within the intervention would be less demanding and thus “more age-appropriate” than in normal production. This means that they expected the work would be less physically demanding and less stressful than in normal production via adaptation of time allowances for conducting job tasks at the assembly line, as well as an adaptation of the work environment.

*“You’re not getting any younger, and then I thought, ok, maybe a little more leisurely, I thought, but that's not the case.” (MA8, 115)*

Interviewees reported that the company had advertised for participation in the intervention by claiming that time allowances would be lowered, i.e., participants would have some scope of time for finishing the workflow at their own speed. However, they realized that job demands were not lower than in normal production and got disappointed. The interviewees reported that for this reason some workmates abandoned their participation in the intervention and returned to normal production.

Interviewees formulated expectations of the intervention that were closely related to their general expectations of the company´s dealing with older workers.

Starting from the assumption that older workers have decreasing physical capacities, what, makes some activities harder for them to execute, interviewees expressed several expectations towards the company. First, they expected the company to create ergonomically designed workplaces that facilitated work for older workers by demanding, e.g., less strength or different movements. Second, they expected the company to set time allowances for older workers in a way that allowed them to finish a workflow without stress. Third, interviewees expected the company to provide a functioning job rotation, as this was considered more important for older workers, and, fourth, to offer work to older workers that does not overburden them mentally or physically. It became clear that despite workers` individual differences in physical capacities, they all identified the same work tasks, namely those involving lifting, pushing, kneeling, and bending, as demanding for older workers, particularly for those with impairments.

Beyond that, the meaning of work for some interviewees became clear when they were asked to think about the effects of the relocation they experienced due to their participation in the workplace intervention. To most, work mainly meant financial security: they needed to bridge the time until retirement and were happy to be able to earn money somehow. Thus, they attempted to fulfill job demands despite their own physical limitations. Against this background, interviewees´ work attitude can be described as pragmatic. This is also expressed by the following citation:

*“Uh, I have to decide, if I don't have that, what kind of alternative do I have? At my age none at all, yes. That's the way it is. If I was fired today, or- or make the change somewhere else outside, I have no chance, with my qualifications and at [the age of] 60. “(MA6, 181)*

Interviewees also placed high value on a good working atmosphere and a good relationship with colleagues.

### Management II: implementation of the intervention evoked perceptions of a lack of being valued and supported by the company as well as feeling of insecurity

The company´s handling of participants´ complaints about problems with the implementation of the workplace intervention in workers provoked a feeling of not being supported and not being valued by the company. This perception was due to two main characteristics of the company´s management of the implementation.

First, the company was perceived to ignore workers´ complaints about occurring problems and proposed improvements. This concerned the malfunction of job rotation due to personnel shortages, as well as workers´ problems with time allowances and design of the work environment. Workers reported that they had complained about problems with time allowances, yet these were not adapted until October 2014 when the interviews took place. Workers perceived that management and work council were not interested in their constant problems, and felt left alone with these problems. Thus, interviewees assessed the company´s work council as advocating the company´s interests more than workers´ interests:

*“Works councils themselves, yah, they’re paid by employers, right. They have to do what they are doing at the moment. […] Because- he [the member of the work council] had already been called a couple of times that he should come and have a look at the [age-appropriate workplaces] too, and- and he never came. He has no interest at all or claims he has no time.” (MA6, 203)*

The feeling of being left alone with one´s problems was increased by the fact that the group spokesmen was responsible to mentor teams twice the size as in normal production, implying that he had little time to communicate with workers. Second, the company was perceived as not willing to invest in the effective implementation of the intervention. This perception was ascribed to the fact that the work environment was outdated and little investments were made to improve ergonomics to the needs of older workers as the following citation illustrates:

*“Ergonomically speaking or so, some things may be better in mass production, because you simply spend money on it.” (MA7, 87)*

Workers perceived the company´s unwillingness to invest in an effective implementation as a signal of being of little value to the company, and the intervention of being of little importance.

In addition, the implementation of the workplace intervention with an uncertain time horizon evoked a feeling of insecurity about future occupational perspectives inside the company. Several workers expressed that working in the production of a limited edition of a product made them fear that they might be forced to leave the company even before regular retirement age, as the company might have no need for them after the production of the limited edition would be completed.

## Discussion

As outlined above, older workers perceived the workplace intervention and its implementation largely as negative. From the perspective of participants, the intervention did not facilitate their work, but even created new challenges. The fit between work requirements and individual capacities was not improved. Hereafter, some reflections are provided on how each of these challenges could have been prevented (see also Table 4 in the supplementary file). For this purpose, findings are compared to evidence from the literature.

*First,* the participation in the intervention created physical challenges for older workers as work environment and work equipment of the intervention did not match their physical capacities. The problems they reported (lifting objects, strength, repeated movements and temperature of the production hall) can be understood by reference to findings from the literature on physical capacities of older workers.

As studies show, older workers suffer from a general deterioration in muscle strength (Ostchega et al. 2004) and are more likely to have injuries or MSD (De Zwart et al. 1996), leading to a limited ability to lift heavy objects or exert repeated movements over longer periods of time (Chan et al. 2000). Furthermore, hand grip strength (Dodds et al. 2014), agility (Fujiyama et al. 2012; Kenny et al. 2008; Marques et al. 2016; Ostchega et al. 2004), eyesight (Chan et al. 2000; Silverstein 2008), heat tolerance (Kenny et al. 2016b; Lamarche et al. 2017; Larose et al. 2013; McGinn et al. 2017; Stapleton et al. 2015) and cold tolerance decrease with age (Blatteis 2012). However, there are individual differences as factors such as physical activity and lifestyle have an impact on the deterioration of physical capacities (Leyk et al. 2009, 2010).

Also, older workers reported problems with regard to performing complex motion sequences implied by working at a standing assembly line, without hydraulic lifts and with no possibility to fit the height of the assembly line to body size. These problems are in line with findings from the literature. As studies point out, older workers might have problems with the execution of complex motor tasks requiring coordination or integration of multiple large muscle tasks due to negative muscle recruitment that impacts muscle coordination and balance (Fujiyama et al. 2012; Marques et al. 2016). A mismatch between work demands and physical capacities of older workers increases the risk of injury (Chan et al. 2000; Fraade-Blanar et al. 2017; McCaughey et al. 2013; Pransky et al. 2005).

With regard to workplace adaptation, ergonomic workplaces can support older workers´ productivity and work ability if they are age-appropriate (Poscia et al 2016). Problems reported by older workers suggest that ergonomic adaptations of the workplace did not take place or at least did not involve the overall work environment. However, findings suggest that the general work environment of a workplace intervention should be adapted to the needs of older workers as well, e.g., by introducing air conditioning, as the climate might impact older workers´ performance and well-being. Thereby, physiological stress caused by the work environment could be reduced. For age-appropriateness of workplaces, it might be helpful to let older workers participate in the design, as has been proven to be effective for younger workers (Rivilis et al. 2008).

*Second,* the intervention demanded from participants to learn new, long sequences of tasks as implied by the longer work cycle, yet little training was provided. Some workers perceived the learning of long sequences as challenging. This finding is in accordance with evidence provided by the literature. It has been shown that ageing might have a negative impact on the working memory and the cognitive processing speed which are important for learning performance (Fisher et al. 2017). Thus, it might be more difficult for older workers to learn new and difficult tasks and to recall previously learned information during task performance (Salthouse & Babcock 1991). This is particularly the case when learning of new or complex tasks occurs under time pressure (Fisher et al. 2017).

For workplace interventions, this implies that cognitive capacities should be adequately considered when designing work processes for older workers in industrial production. In addition, training should be adapted to their needs, e.g., by planning additional time for training. Also, self-paced training might have a positive effect on older workers´ training performance of older workers (Callahan et al. 2003), as well as allowing them to self-select their learning strategies (Fisher et al. 2017). Prevention of cognitive decline could also be an appropriate approach for companies, e.g., by offering training or assigning cognitively demanding and alternating job tasks for blue collar workers even at a younger age. This might help to sustain workers´ cognitive capacities until retirement.

*Third,* older workers experienced stress due to preset time-allowances for work sequences which they perceived as too tight. As Morschhäuser (1999) suggests, time-allowances might be a general strain for older workers as they cause stress. Also, older workers show greater physiological stress in response to high job demands (Ritvanen et al. 2006) and tend to take longer to recover (Kiss et al. 2008). However, work stress is negatively associated with work ability of workers above the age of 50 (Yang et al. 2019). Hence, stress caused by preset time-allowances might have counteracted the objective of promoting work ability.

To prevent stress, flexible time-allowances that allow workers to finish their work at their own speed could be helpful. Flexible time-allowances would also be advantageous as they allow to account for individual differences with regard to individual capacities of older workers. As an alternative, a participation of older workers in setting time allowances for work sequences could help to determine adequate job requirements, as has been suggested by interviewees.

Furthermore, older workers could benefit from selection, optimization, compensation (SOC) training to learn how to deal with job demands. SOC training has been found to positively impact (perceived) work ability (Müller et al. 2013; Weigl et al. 2013) and can be implemented in the framework of workplace interventions (Müller et al. 2016).

*Fourth,* job rotation offered little physical relief to workers. Personnel shortages hindered rotation and work stations did not differ much with regard to motion sequences. This malfunction of job rotation had a negative effect on workers´ reported satisfaction and presumably a negative effect on work ability as well.

Existing literature finds a positive impact of job rotation, i.e., a change among tasks that requires the use of different body parts, on perceived job satisfaction and perceived productivity (Van Wyk et al. 2018; Jeon et al. 2016). However, findings of studies examining the effect of job rotation on musculoskeletal complaints, MSD prevention and work ability are inconsistent, as shown Leider et al. (2015). With regard to improving workplace interventions, findings imply that improvement of job rotation could at least improve workers´ satisfaction. Thus, personnel shortages should be scheduled and prevented, e.g., by including younger workers into mixed-age teams instead of implementing production only with older workers. In addition, work processes should be designed in a way that real job rotation is possible, i.e., job demands at different work stations should implicate the use of different body parts, whereby individual work-related impairments can be considered. For this purpose, a short-term staff planning system which takes into account workers´ qualifications and recent allocation as proposed by (Hochdörffer et al. 2017) might be helpful.

*Fifth,* participants in the intervention perceived it as a major challenge that they had to leave their usual work environment and particularly their colleagues. Relatedly, they had problems to adjust to new work conditions imposed by the workplace intervention.

This finding is in line with findings from other studies which show that older workers might have more difficulties to adjust to new jobs and conditions at the workplace than younger workers (Poscia et al. 2016). Also, older workers might rather choose to retire than to adapt to changes (Yeatts et al. 2000). Thus, when designing workplace interventions consideration should be given what changes a participation implies for participants. It seems to be a good idea to limit the amount of changes with regard to work environment, colleagues and job tasks workers are facing due to their participation in a workplace intervention, and to offer social support in the process of change. In addition, selection, optimization, compensation (SOC) training might help older workers to adapt to a changing workplace (Ng and Law 2014) and have a positive impact on their work ability (Müller et al. 2013, 2016). Work community can also play an important role in supporting work ability (Ilmarinen 2009). Thus, it might be reasonable to undertake workplace interventions for existing teams.

*Sixth*, the design of the workplace intervention created a work situation which invoked age stereotypes. The intervention originally included only older workers and workers with impairment. As older workers felt over-challenged with work requirements, they felt the need to distance themselves from their age group. Thus, they tried to avoid being assessed as not full-value worker due to their age. This was particularly the case when younger workers worked temporarily in the production hall to compensate for personnel shortages. Older workers´ behavior can be interpreted as a stereotype threat effect, i.e., a behavior that occurs when an individual feels at risk of confirming a negative stereotype about their group, which often occurs in regard to aging at work (Lamont et al. 2015; Isopahkala-Bouret 2015). As studies show, stereotype threat can discourage older workers, increase absenteeism, lower retention, undermine their performance, have a negative effect on their health in general (Lamont et al. 2015) and their episodic and working memory performance in particular (Armstrong et al. 2017). Thus, the design of the workplace intervention might have negatively affected participants´ work ability.

To avoid stereotype threat effects, companies can apply age conscious policies and develop a workplace culture that embraces older workers´ strengths. In general, this means that companies account for the heterogeneity of circumstances and needs of their workers and improve the work conditions for all as suggested before (Cloostermans et al. 2015; Poscia et al. 2016). With regard to the design of workplace interventions, the heterogeneity of the workforce and resulting disparities in work performance should be addressed in different subgroups of workers which are not defined by chronological age (Loeppke et al. 2013; Poscia et al. 2016). Thus, a spatial separation of older and younger workers could be avoided.

With regard to the improvement of workplace culture, different efforts can be made. As a study suggests, trainings for workers are effective in altering attitudes and reframing cognitions of older workers by providing knowledge on how ageing impacts on work ability and needs (Skoglund and Skoglund 2005).

*Seventh,* workers felt badly informed about the modalities of the intervention and their expectations of the intervention were disappointed. This negatively impacted their satisfaction with the intervention and their attitude towards the company. Thus, management of the intervention might have had a negative impact on work ability promotion.

To improve participants´ satisfaction with workplace interventions, companies should a priori better inform their workers about the modalities of the intervention. Also, as studies on work health promotion for workers from all age groups suggest, workers´ participation in the process of designing health interventions of different kinds can render them more effective and can improve workers´ satisfaction (Conn et al. 2009; Rivilis et al. 2008). This might apply for workplace interventions for older workers as well. Participation can have different forms as Abildgaard et al. (2018) outline with regard to work environment interventions which seek to improve workers´ health or well-being.

*Finally,* participants´ attitude towards the company was affected by the way the intervention was implemented. They did not feel supported by the company due to low investments of the company into improving the implementation of the intervention. As the intervention was implemented spatially separate from normal production and the production line had a limited time frame, workers felt insecure about their future career inside the company.

Dhar (2012) shows that the perception of low organizational support by a company negatively impacts workers attitudes towards the organization. Also, a constrained occupational time perspective is negatively related to older workers´ learning self-efficacy (Kochoian et al. 2017). Thus, the design and implementation of the intervention might have negatively affected older workers´ individual capacities and work ability.

To improve the implementation of interventions, companies could invest in job and work environment design, thus signaling participants in workplace interventions that they are valued. Also, companies can communicate an occupational perspective to foster workers´ motivation. Furthermore, lending workers an ear for complaints with regard to the implementation of the intervention could signal support. To that end, the conduct of a process-accompanying evaluation of the intervention that includes workers´ perspectives and allows the intervention to be adapted promptly might be helpful.

In summary, it can be assumed that the workplace intervention could have been more effective with regard to promoting the work ability, if the challenges that older workers identified would have been prevented. For this purpose, companies could have undertaken different kinds of actions.

### Strengths and limitations of the study

The qualitative design of the study chosen was useful, as it allowed to explore older workers´ experiences and expectations of the workplace intervention.

A limitation of the study is the small sample size and that only older workers participating in the workplace intervention were interviewed. As the company withdrew consent for the conduct of these interviews, neither a company doctor, a member of the workers´ council or a person responsible for the intervention were interviewed. An independent assessment of work environment, work equipment or work organization inside the intervention might have come to different conclusions than participants themselves. Also, all participants were male, reflecting the predominant gender ratio inside in the intervention. A female participant might have raised further or different issues with regard to challenges arising from the implementation of the workplace intervention, as older female workers might have different needs and perceptions than older male workers.

Furthermore, the findings reflect work conditions and the organizational culture of only one industrial company. However, findings might be relevant for the successful implementation of workplace interventions for older workers in general.

In addition, all participants in the study were approached via responsible persons for the measure which possibly could imply a bias with regard to the assessment of the workplace intervention. Yet, as the participants in the study were frank in raising a number of criticisms, the method of sampling or the problem of socially desirable responses did not seem to play a role for the assessment of the workplace intervention.

## Conclusion

This paper set out to depict older workers´ expectations and experiences of a workplace intervention they participated in. It was motivated by the assumption that including participants´ perspective can help to render workplace interventions more effective with regard to the promotion of work ability. As findings from the study show, the design and implementation of the workplace interventions created new challenges for participants which presumably counteracted its intended objective of promoting work ability. For example, physical challenges for older workers created by a low degree of automation and the lack of air-conditioning in the production hall evidently did not promote their work ability.

Comparing findings from the study to evidence on aging and work ability provided by the literature, we provided some reflections on how design and implementation of workplace interventions could be improved. As becomes clear from the discussion, the company could have prevented the occurrence of some of the challenges arising for older workers from their participation in the workplace intervention. For this purpose, two approaches seem obvious: first, to refer to scientific evidence on physical and cognitive capacities of older workers, second, to include older workers when planning the design and implementation of a workplace intervention. Presumably, these two approaches could improve future workplace interventions implemented by companies to sustain and promote the work ability of older workers as well.

## Supplementary Information

Below is the link to the electronic supplementary material.

Supplementary file1 (DOCX 36 KB)

## Data Availability

The datasets used and/or analyzed during the current study are available from the corresponding author on reasonable request.
