# Supplementary material for: Challenges arising for older workers from participating in a workplace intervention addressing work ability: a qualitative study from Germany
Source: Int Arch Occup Environ Health. 2021 Jan 24;94(5):919–33. doi: 10.1007/s00420-020-01639-x (PMC8238737; doi:10.1007/s00420-020-01639-x)
Supplement: Supplementary file 1 — Supplementary file1 (DOCX 36 KB) [file 420_2020_1639_MOESM1_ESM.docx]

Appendix

Table 1 Sample of interviewed employees

| Characteristic | Number |
| --- | --- |
| Sex  Male  Female | N=8  N=0 |
| Age at time of interview (ø) | 53.75 years |
| Number of years in company at time of interview (ø) | 26.25 years |
| Highest educational achievement |  |
| Secondary school qualification | 2 |
| Completed apprenticeship | 5 |
| Unknown qualification | 1 |

Table 2 Main questions of the interview guide for participants in the workplace intervention

| Topic | Question |
| --- | --- |
| Work life | Could you please outline your working life, starting from apprenticeship?  What was your last job inside the company? |
| Workplace intervention | How did you learn about the intervention?  What was your motivation to participate in the intervention?  What did you expect of the intervention? To what extent were your expectations met?  How did you experience transfer to the intervention?  Which changes did the participation in the intervention imply for you?  What is your current job?  How do you assess your decision to participate in the intervention now? |
| Work integration | How does cooperation in the intervention differ from cooperation in normal production?  How do you experience cooperation between younger and older workers?  To what extent are your (physical/psychological) needs considered by the company?  How do you experience managers´ attitudes towards older workers?  To what extent does the company differentiate between younger and older workers?  To what extent are needs of older workers considered by the company? |
| Individual abilities and needs | What job tasks do you experience as difficult?  To what extent do your age or physical and psychological changes affect your work?  How do you assess your own work capacity? |
| Personal beliefs | What are older workers´ strengths that the company can benefit from?  What does work mean for you?  What does age-appropriate work mean for you? What does it look like?  What do you think about generation management? |

Table 3 Overview of challenges arising for older workers from participating in the intervention

| Challenge for older workers | Problems related to the intervention as perceived by older workers |
| --- | --- |
| Work environment and work equipment: physical challenges arising from low automation and outdated work environment | Outdated equipment, low degree of automation  No possibility to fit the height of the assembly line to body size, no hydraulic lifts  Need to move more: complex motion sequences and long distances  Small production hall  Insufficient illumination, no air conditioning |
| Work process design: cognitive challenges arising from the introduction of a long work cycle | Learning of new, long sequences of tasks with insufficient initial training  Prolongation of work cycle: longer work sequences to remember and higher responsibility for correct execution of tasks |
| Work organisation I: increased job demands induced by tight time allowances | Time pressure due to preset tight time allowances  Little time for recovery between work sequences and cycles |
| Work organisation II: low possibility for recovery due to a malfunction of job rotation | Personnel shortage, exacerbated by sick leaves  Workers with impairments working at limited number of work stations  Little variety of demands between work stations |
| Work organization III: change of teams and social status | Change of teams  Change of social positions due to giving-up of former positions such as group spokesman or supporter |
| Work organization IV: need for counter-acting age stereotypes invoked by the design of the workplace intervention | Participation in intervention only for older workers and workers with impairments  stereotype threat caused: younger workers compensating for personnel shortages caused older workers to distance from their age group |
| Management: bad information about the intervention causing disappointment | Older workers´ expectations disappointed: not lower physical demands and not less time pressure than in normal production |
| Management: implementation of the intervention evoked perceptions of a lack of being valued and supported by the company as well as feeling of insecurity | No reaction to workers´ complaints about the implementation of the intervention  Little investment in improving the work environment  Limited time frame of the production line and separation caused occupational insecurity |

Table 4 Overview of recommendations to avoid challenges

| Problems related to the intervention as perceived by older workers | Recommendations |
| --- | --- |
| Outdated equipment, low degree of automation  No possibility to fit the height of the assembly line to body size, no hydraulic lifts  Need to move more: complex motion sequences and long distances  Small production hall  Insufficient illumination, no air conditioning | Take ergonomic adaptations of the workplace as well as the general work environment to reduce physiological stress  Include older workers in designing an age-appropriate workplace |
| Learning of new, long sequences of tasks with insufficient initial training  Prolongation of work cycle: longer work sequences to remember and higher responsibility for correct execution of tasks | Provide age-appropriate training for new tasks, e.g. plan additional time for training, allow self-paced training and/or self-selection of learning strategies  Consider cognitive capacities of older workers when designing work processes  Prevent cognitive decline of workers by alternating tasks etc. |
| Time pressure due to preset tight time allowances  Little time for recovery between work sequences and cycles | Implement flexible time-allowances to decrease stress or let workers participate in setting time allowances  Provide SOC training on how to deal with job demands |
| Personnel shortage, exacerbated by sick leaves  Workers with impairments working at a limited number of work stations  Little variety of job demands between work stations | Prevent personnel shortages to secure the functioning of job rotation  Design work such that job demands implicate the use of different body parts at different work stations |
| Change of teams  Change of social positions due to giving-up of former positions such as group spokesman or supporter | Limit amount of changes implied by participating in a workplace intervention, e.g. by implementing workplace interventions for existing teams  Provide social support in the process of change  Provide SOC training to facilitate adaptation to new workplace |
| Participation in intervention only for older workers and workers with impairments  stereotype threat caused: younger workers compensating for personnel shortages caused older workers to distance from their age group | Improve work conditions for all workers according to their needs  Design workplace interventions that address subgroups of workers which are not defined by chronological age  Improve workplace culture by offering trainings which alter attitudes and reframe cognitions of older workers |
| Older workers´ expectations disappointed: not lower physical demands and not less time pressure than in normal production | Improve provision of information about modalities of workplace intervention  Include older workers in design of workplace interventions |
| No reaction to workers´ complaints about the implementation of the intervention  Little investment in improving the work environment Limited time frame of the production line and separation caused occupational insecurity | Consider workers´ complaints with regard to implementation of the intervention  Improve the implementation promptly  Provide occupational perspective |

Table 5 Reporting the study referring to the COREQ 32-item checklist

| Domain 1: research team and reflexivity |  |
| --- | --- |
| *Personal Characteristics* |  |
| Interviewer/facilitator | The interviews were conducted by Pia Schmalzried as part of her master thesis in sociology. These interviews were re-analysed for the present study. |
| Credentials | Susanne Völter-Mahlknecht - MD  Monika A. Rieger – MD  Khira Sippli – M.A.  Pia Schmalzried - M.A. |
| Occupation | At the time of the present study, Susanne Völter-Mahlknecht and Monika A. Rieger were professors at the Institute of Occupational and Social Medicine and Health Services Research, University Hospital of Tuebingen.  Khira Sippli was occupied as research associate at the Institute for Applied Economic Research at the University of Tuebingen.  Pia Schmalzried was working in human resources in the private sector. |
| Gender | The researchers are female. |
| Experience and training | Susanne Völter-Mahlknecht has a professional background in Occupational Medicine.  Monika. A. Rieger has a professional background in Occupational Medicine.  Khira Sippli has a background in Sociology and Political Science.  Pia Schmalzried has a background in Sociology. |
| *Relationship with participants* |  |
| Relationship established | The interviewer conducted a participating oberservation of the production, prior to the interviews. However, with participants no relationship was established before conducting the interviews. |
| Participant knowledge about the interviewer | The participants were informed that the interviewer was a graduate student in Sociology and conducted the interviews as part of her master thesis on the topic of age management. |
| Interviewer characteristics | See 7. The interviewer met the participants for face-to-face interviews. |
| Domain 2: study design |  |
| *Theoretical framework* |  |
| Methodological orientation and theory | The methodological orientation underpinning the present study was content analysis according to Mayring (2014). |
| *Participant selection* |  |
| Sampling | Convenience sampling was chosen, as there was no direct access to participants in the workplace intervention and interviews were conducted on a voluntary basis. |
| Method of approach | Participants were approached by a person responsible for the workplace intervention from the human resources department of the company. |
| Sample size | Eight participants were included in the study. |
| Non-participation | None. |
| *Setting* |  |
| Setting of data collection | Interviews were conducted face-to-face in a breakroom at the workplace intervention site. |
| Presence of non-participants | No other persons than the participants and the interviewer were present during the interviews. |
| Description of sample | Only 8 participants of the workplace interventions were included in the sample. All participants were male, aged 53 or above and long-term employees of the manufacturing company. |
| *Data collection* |  |
| Interview guide | Themes of the interview guide are provided in table 1 of the appendix. |
| Repeat interviews | No repeat interviews were carried out. |
| Audio/visual recording | Audio recording was used to collect the data. |
| Field notes | Field notes were made during the interviews, yet not included in the study. |
| Duration | The interviews lasted approximately one hour each. |
| Data saturation | Data saturation was discussed in the method section. |
| Transcripts returned | Transcripts were not returned to the interviewees. |
| Domain 3: analysis and findings |  |
| *Data analysis* |  |
| Number of data coders | The data was coded by one researcher. Codings were discussed with a second researcher. |
| Description of the coding tree | No. |
| Derivation of themes | While codes were derived inductively from the material, themes were both identified in advance and derived from the data. |
| Software | MAXQDA 11 plus was used to manage and analyze the data. |
| Participant checking | Participants did not provide feedback on the findings from the study as this was not intended for the master thesis and their contact data were not collected. |
